# A Literature Study of Medical Simulations for Non-Technical Skills Training in Emergency Medicine: Twenty Years of Progress, an Integrated Research Framework, and Future Research Avenues

**DOI:** 10.3390/ijerph20054487

**Published:** 2023-03-02

**Authors:** Cevin Zhang

**Affiliations:** School of Media and Design, Beijing Technology and Business University, Sunlight South Road 1, Beijing 102488, China; chenzh@kth.se

**Keywords:** emergency medicine, communication, leadership, crew resource management, high-fidelity simulation training

## Abstract

Medical simulations have led to extensive developments in emergency medicine. Apart from the growing number of applications and research efforts in patient safety, few studies have focused on modalities, research methods, and professions via a synthesis of simulation studies with a focus on non-technical skills training. Intersections between medical simulation, non-technical skills training, and emergency medicine merit a synthesis of progress over the first two decades of the 21st century. Drawing on research from the Web of Science Core Collection’s Science Citation Index Expanded and Social Science Citation Index editions, results showed that medical simulations were found to be effective, practical, and highly motivating. More importantly, simulation-based education should be a teaching approach, and many simulations are utilised to substitute high-risk, rare, and complex circumstances in technical or situational simulations. (1) Publications were grouped by specific categories of non-technical skills, teamwork, communication, diagnosis, resuscitation, airway management, anaesthesia, simulation, and medical education. (2) Although mixed-method and quantitative approaches were prominent during the time period, further exploration of qualitative data would greatly contribute to the interpretation of experience. (3) High-fidelity dummy was the most suitable instrument, but the tendency of simulators without explicitly stating the vendor selection calls for a standardised training process. The literature study concludes with a ring model as the integrated framework of presently known best practices and a broad range of underexplored research areas to be investigated in detail.

## 1. Introduction

Recent advances in the application of non-technical skills in emergency medicine have led to a growing interest in the use of medical simulations. The integration of medical simulations is an important step in the curriculum development process [1]. Although previous studies have examined simulation modelling in nearly all healthcare systems [2], few studies have adapted the definition of simulation to a fast-paced, team-oriented speciality. This article defines medical simulation in emergency medicine as the educational modality to provide participants with hands-on experiences in the diagnosis and treatment of acute illnesses such as trauma and injuries. Thus far, the area of application has almost entirely focused on eliminating disparities between theory and clinical practice. Realistic medical education uses a wide range of teaching techniques, such as dummies [3], mannequins [4], organs, film and television teaching materials [5,6], simulation cases [7], real patients or simulated patients [8], and so on. Although patient scenarios and conditions could be revised based on the instructor’s choices, a realistic situation of doctors and nurses requires a methodical approach. Utilizing a variety of subspecialties, high-level digital simulation dummies, clinical teaching plans for emergency and severe cases, teamwork training, and scene extension training of various team composite situations allows for the most authentic representation of doctors and nurses in challenging medical settings, improving trainees’ clinical treatment abilities.

Non-technical skills affect quality and safety in emergency medicine. On one hand, frequent adverse events occur often during health monitoring, triage, patient hand-off, and emergency management, indicating a lack of cognitive, social, and personal capacities. Non-technical skills training, on the other hand, is often understudied in comparison to technical skills training, and training outcomes vary between simulation studies. Furthermore, the management of quality and safety care requires certain skill sets such as communication [9], cooperation [10], leadership [11], decision-making [12], coordination [13], collaboration [14], and others [15,16]. These considerations call for more realistic scenarios than those hitherto employed in medical education. In addition, the use of scientific evidence and statistical methods can support formal assessments and evaluations. While the use of information technology is on the rise, there are opportunities and challenges for cutting-edge user interface design. 

The majority of professions specialising in emergency medicine could be actively involved in centre-based simulations with larger facilities or pilot test activities taking place on the premises. Whilst the Tea Bag Model was applied in the early days [17], medical students were required to join a specialist department, spend around three to five years of study, and pass a specialist examination before becoming an attending physician. Later, the concept of competency-based medical education was proposed by the World Health Organization to pinpoint the intended output as a health professional who can practise medicine at a defined level of proficiency [18]. The tendency towards a competency-based approach, which serves as the foundation for a new accreditation model [19], has attained popularity in medical education. Recently, research has given rise to the confirmation of experiential learning theory [20] and flipped learning styles [21,22]. Nevertheless, there has been little discussion about the opportunities that medical simulation provides for non-technical skills training in emergency medicine, as to which non-technical skills can be trained, what types of simulators are available, and which analytical methods are required to process data collected from the operational clinical environment.

Most research on non-technical skills has focused on patient outcomes and how reactions from participants, often through questionnaires, contribute to the effectiveness and usefulness of the instrument [23,24]. There is no overview of training experience design that accounts for the collection of behavioural data and the achievement of intended training goals from the instructor’s perspective. This is arguably due to the research scope of application-oriented studies being mostly constrained to the relationship between professional roles, parameters of the medical simulation category, and details of the investigated patient scenarios. Previous literature studies focused on a single type of medical simulation, publications from a short time period, single research methods, and studies that did not provide comprehensive coverage of non-technical skills, not to mention the level of the operational clinical environment and medical simulation vendors endorsing top-notch research in this field.

This study is a continuation of the work by Zhang et al. [25], with an emphasis on identifying available forms of medical simulation as means for training non-technical skills in emergency medicine. These skills do not require an outstanding technical background; rather, their acquisition and application represent one of the greatest challenges encountered by medical education nowadays. It is, therefore, worthwhile to distil from a comprehensive list of publications the medical simulation categories, involved professions, research methods, specific areas of non-technical skills, behavioural data sources, operational clinical environment, and vendor. The underlying relationships between educational morality, participants, and the instructor are thoroughly examined, resulting in an integrated framework which can be used as a blueprint for other simulation projects. Based on these findings, the objective of this paper is to provide a literature study to answer the following research question:

- What lessons have been learnt over the past two decades from utilizing medical simulations for non-technical skills training in emergency medicine?

## 2. Research Method

The electronic data search draws on the Science Citation Index Expanded (SCI) and Social Sciences Citation Index (SSCI) editions of the Web of Science Core Collection. These editions are the most prominent academic volumes where articles are originally published in significant and high-quality scientific journals. A combined search of the terms “interprofessional,” “leadership,” “teamwork,” “team-work,” “communication,” “decision-making,” “coordination,” “management,” and “non-technical skills” is performed in conjunction with “medical simulation” and “emergency medicine”. “Topic” as a field tag is introduced to the query to narrow down the focus of the literature by recognising matches only within the title, abstract, and keyword sections. All searches are restricted to journal articles with peer review. 

Articles are examined to shortlist relevant literature according to the inclusion criteria. The research should strive to improve non-technical skills or promote the efficiency of non-technical skills training for emergency medicine personnel. Furthermore, participants must have the same level of experience in medical simulations. Abstracts, conference proceedings, posters, book reviews, and entries without access to the full texts were discarded. In addition to these criteria, the paper must fall into any of the following categories:

- Application paper. The study incorporates at least one medical simulation category and implements a simulation-based experiment to illustrate facets of emergency medicine.
- Methodological contribution. The study precedes a simulation of any medical system; rather, it offers either a methodological critique or an evaluation of relevant simulation experiments.
- Literature review. The study synthesises the significant aspects and outcomes from a vast number of references in the investigated area of research.

CiteSpace, an information visualisation tool for bibliometric analysis [26], is used to attribute publications by referencing networks. This presentation lines up scholarly patterns in scientific literature. The advancement of research avenues can be represented by sociogram-like clusters. These clusters consisted of nodes and link lines, respectively, denoting article data and co-occurrence relationships. The visualisation parameters are configured to prioritise significant referencing networks with the clearest labels: time slicing (from 2001 to 2020, years per slice = 1), node type (one selection at a time from keyword and institution), and pruning (none).

## 3. Results

Based on the SCI and SSCI literature, 298 out of 887 papers fulfil the inclusion criteria. Table 1 presents the categorization of 215 application papers, 50 methodological contributions, and 33 literature reviews, and published between 2001 and 2020. Of the entire selection, 161 records account for communication, 101 for teamwork, 68 for leadership, 47 for decision-making, 21 for coordination or collaboration, and 50 for other specific categories.

### 3.1. Medical Simulations for Non-Technical Skills Training in Emergency Medicine: Overview

In recent years, high-fidelity simulation has emerged as the most frequently deployed medical education instrument for enhancing the development of non-technical skills in emergency medicine. Their human–computer interaction interfaces bring together medical practitioners with convenience and fidelity. Among relevant publications, 69 records account for communication, 48 for teamwork, and 29 for leadership. The number of papers adopting mixed-method approaches and utilising observations outnumbers those employing other research methods and data types, respectively. Most simulations were implemented at the unit level with Laerdal’s SimMan products as the most relied-upon items. Schulte-Uentrop et al. presented a correlation between motivation and non-technical skill performance based on Resusci Anne from Laerdal Medical [15], confirming the suitability of the medical simulation used for training a wide range of resuscitation skills. Su et al. deployed the SimMan 3G simulator from Laerdal to strengthen communication in advanced cardiac life support [27]. Through applying an effective communication strategy, the authors demonstrated the positive effects of high-fidelity simulation on self-efficacy and knowledge transfer. Depending on the adequacy of the solution, the instructors could resemble high-fidelity environments via the process of integrating human–patient simulations [28], mannequins [29], or simulation suites [30] to work with one another.

Human–patient simulations, which model virtual appearances and behaviours of high relevance for the context of the planned simulation activity, are promising interactive learning systems across specialities, particularly emergency medicine. Full-responsive models with realistic characteristics could display neurological, physiological, or physical symptoms. Implementing computerised or pre-programmed scenarios would engage training participants in communication and teamwork. Among publications, 39 records account for communication, 25 for teamwork, and 21 for leadership. Observations and mixed-method approaches were used in most articles. The majority of simulations were carried out at the unit level with Laerdal’s SimMan product as the most popular item among the wide range of wares. To disseminate innovations in medical education, a central venous catheter simulator has been successfully adopted to incorporate the expertise of internal and emergency medicine residents in different medical education settings [31]. This study underlined the active role of educational leadership in the administration of medical simulation programmes. Armbruster et al. showcased 25 exemplary missions carried out by emergency doctors in a training programme driven by NASimSaar25 [32], a patient simulator well evaluated by the majority of the target group. Simulation training prepared participants for rare emergencies and invasive measures. Stone et al. proposed a standardised setup consisting of SimBaby from Laerdal and the resuscitation room of the emergency department [33]. The curriculum format was considered the best arrangement for resuscitation education on the virtue of leadership and communication.

Mannequin-based simulation is based on instrumented human models that differ in major areas of styles, personalities, and aspirations. The physiological motivation provided by a whole-body component in conjunction with a variety of accessories allows for in-depth training of a wide range of non-technical skills. Among publications, 28 records account for communication, 14 for teamwork, and 13 for leadership. The numbers of articles using observations, mixed-method approaches, and unit-level settings outweigh all other categories by a large margin. SimMan and Resusci Anne from Laerdal were the prevalent simulation products. Dadiz et al. deployed Noelle and HAL from Gaumard Scientific as mother and infant mannequins in a simulation-based, interdisciplinary training programme [9]. Results showed that providers’ communication in the delivery room and with families improved over time. Dermody et al. utilised a Fiberoptic Evaluation Station, including task trainers, to develop an intensive simulation-based course [34]. More than 85% of the participants confirmed the usefulness of the intervention in helping them possess appropriate knowledge and increase their self-confidence. Simulated Emergency Medical Service missions were launched using Ambu Mini MegaCode from Ambu A/S Ballerup, according to Zimmer et al.’s study [35]. Through video-based observations, the authors successfully identified unwarranted behaviours that would jeopardise patient safety, a process in which mannequins may serve as overall facilitators with a focus on the quality of communication during actual deliveries of medical care.

In situ simulations can be useful to uncover active errors and identify salient aspects of the environment at the place of occurrence. Participants are repeatedly exposed to contextual data via hands-on workshops, which provide more information for understanding the relationship between individual practice and the larger medical system. Twenty-three records account for communication, 21 for teamwork, and six for leadership. It is worthwhile to note the prevalence of quantitative methods. Additionally, there is an equal number of studies performing simulations at the unit and department levels. Laerdal’s SimMan products, however, remain the top choice. West et al. conducted interprofessional simulation activities using a disaster-day instrument [36]. The findings revealed higher students’ self-ratings on average than those of the standardised patients. Couto et al. compared teamwork in emergency departments using video-based assessments [37]. On the basis of survey results, in situ simulation was considered more realistic and effective. To facilitate consistent safety measures during airborne rescue missions, Lischke et al. used MedSim to exercise communication, decision-making, and situational awareness [38]. Their pilot project introduced rich contextual information to the overview of the emergency scenario using terrain and helicopter wagons inside a mountain rescue training centre. 

Screen-based simulations in emergency medicine are increasingly being recognised as multimedia systems with human–computer interfaces. It has the potential for human–computer interfaces to curate graphical features, texts, and animations referring to illnesses or injuries as needing immediate medical attention. They have demonstrated outstanding efficacy by virtue of their built-in analytical tools, particularly computing systems, to understand the behavioural patterns of participants in a simulated environment, which is valuable information for the activation of design features that are relevant to the training experience. Among publications, five records account for communication, three for leadership, and two for decision-making. There is a greater number of articles using mixed-method approaches. However, outcomes as a data source and simulations at the unit level attract the fewest applications. Gerard et al.’s simulation received favourable reviews for involvement, realism, and instructional value [39]. Results showed practical evidence for serious game scores to formally assess knowledge and skills in emergency medicine. Through online commercial games, Greci et al. recreated a community-based hospital and its surrounding neighbourhood for training skills in communication and decision-making processes during catastrophic events [40]. All participants reported positive experiences and learned more about disaster preparation. In order to practise teamwork in disaster management, Luigi Ingrassia et al. employed the Interactive Simulation Exercise for Emergencies Simulator to showcase the logistical aspects of emergency service planning [41]. This computer simulation proposed an innovative step in the efficient delivery of the disaster medicine curriculum.

**Table 1:** Classification of papers.

Research Focus	Reference	Non-Technical Skill	Stated Participant’s Profession	Research Method	Data Collection Sources	Respective Operational Clinical Environment	Medical Simulation Product and Vendor
High-fidelity simulation	[10,12,15,23,27,28,29,30,34,42,43,44,45,46,47,48,49,50,51,52,53,54,55,56,57,58,59,60,61,62,63,64,65,66,67,68,69,70,71,72,73,74,75,76,77,78,79,80,81,82,83,84,85,86,87,88,89,90,91,92,93,94,95,96,97,98,99,100,101,102,103,104,105,106,107,108,109,110,111,112,113,114,115]	Communication (69); Teamwork (48); Leadership (29); Decision-making (19); Coordination/collaboration (12); Others (3)	Residents (26); Nurses (24); Physicians (17); Students (15); Doctors (6); Midwives (5); Therapists (5); Paramedics (4); Assistants (3); Gynaecologists (3); Surgeons (3); Anaesthesiologists (2); Pharmacists (2); Technicians (2); Trainees (2); Others (13)	Mixed-method (42); Quantitative (27); Qualitative (9); n/a (5)	Observational (45); Outcomes (24); n/a (20)	Unit (46); Department (17); Institution (16)	SimMan, Laerdal (10); SimMan 3G, Laerdal (5); METI, Medical Education Technologies (4); Resusci Anne, Laerdal (2); HAL, Gaumard Scientific (2); SimNewB, Laerdal (2); SimBaby, Laerdal (2); Noelle, Gaumard (2); SimJunior, Laerdal (1); PAEDSIM, Universitätsklinikum Tübingen (1); PediaSIM, CAE Healthcare (1); UltraSim, MedSim (1); PROMPT birthing simulator, Limbs and Things (1); Others (8)
Patient simulation	[10,11,16,31,32,33,60,62,67,68,84,85,87,95,96,97,105,107,110,112,114,115,116,117,118,119,120,121,122,123,124,125,126,127,128,129,130,131,132,133,134,135,136,137,138,139,140,141,142,143,144,145,146,147,148,149,150,151,152]	Communication (39); Teamwork (25); Leadership (21); Decision-making (12); Coordination/collaboration (5); Others (7)	Residents (16); Nurses (14); Physicians (14); Students (18); Midwives (1); Therapists (3); Paramedics (6); Gynaecologists (1); Surgeons (2); Anaesthesiologists (3); Pharmacists (1); Technicians (1); Trainees (1); Others (9)	Mixed-method (30); Quantitative (17); Qualitative (9); n/a (2)	Observational (35); Outcomes (17); n/a (13)	Unit (33); Department (11); Institution (11)	SimMan, Laerdal (20); SimMan 3G, Laerdal (7); METI, Medical Education Technologies (4); Resusci Anne, Laerdal (4); HAL, Gaumard Scientific (1); SimNewB, Laerdal (2); SimBaby, Laerdal (3); Noelle, Gaumard (1); Resusci Junior, Laerdal (1); CAE Healthcare (1); PROMPT birthing simulator, Limbs and Things (1); Atmosphere, Adobe Systems (2); ECS, Disastermed.Ca (1); CVC simulator, GT Simulators (1); Others (10)
Mannequin	[9,23,34,35,44,45,46,47,87,88,94,96,97,98,99,100,101,102,103,104,105,106,107,108,109,110,111,112,113,114,115,116,117,118,119,120,121,122,123,124,125,126,127,128,129,130,131,132,133,134,135,136,137,138,139,140,141,142,143,144,145,146,147,148,149,150,151,152,153,154,155,156,157,158,159,160,161,162]	Communication (28); Teamwork (14); Leadership (13); Decision-making (6); Coordination/collaboration (1); Others (2)	Residents (10); Nurses (12); Physicians (8); Students (7); Doctors (4); Midwives (2); Therapists (3); Paramedics (2); Gynaecologists (1); Surgeons (1); Anaesthesiologists (1); Pharmacists (1); Obstetricians (3); Interns (2); Others (8)	Mixed-method (28); Quantitative (2); Qualitative (6)	Observational (26); Outcomes (6); n/a (6)	Unit (20); Department (7); Institution (9)	SimMan, Laerdal (12); METI, Medical Education Technologies (1); Resusci Anne, Laerdal (4); HAL, Gaumard Scientific (1); Noelle, Gaumard (2); UltraSim, MedSim (1); PROMPT birthing simulator, Limbs and Things (2); Atmosphere, Adobe Systems (1); MegaCode, Ambu A/S Ballerup (1); Others (4)
In situ simulation	[36,37,38,46,65,66,81,88,103,104,106,107,108,109,111,145,151,163,164,165,166,167,168,169,170,171,172,173]	Communication (23); Teamwork (21); Leadership (6); Decision-making (5); Coordination/collaboration (3); Others (2)	Residents (9); Nurses (19); Physicians (12); Students (3); Doctors (1); Midwives (1); Therapists (6); Paramedics (1); Assistants (3); Gynaecologists (1); Surgeons (2); Anaesthesiologists (4); Pharmacists (4); Obstetricians (2); Technicians (3); Others (9)	Mixed-method (6); Quantitative (10); Qualitative (11); n/a (1)	Observational (19); Outcomes (9); n/a (5)	Unit (10); Department (10); Institution (5)	SimMan, Laerdal (3); SimMan 3G, Laerdal (3); Resusci Anne, Laerdal (1); HAL, Gaumard Scientific (3); SimBaby, Laerdal (1); Noelle, Gaumard (2); PAEDSIM, Universitätsklinikum Tübingen (1); PROMPT birthing simulator, Limbs and Things (1); ECS, Disastermed.Ca (1); SimCapture, B-line Medical (1); Others (3)
Screen-based simulation	[39,40,41,174,175,176,177,178,179,180,181]	Communication (5); Teamwork (1); Leadership (3); Decision-making (2); Coordination/collaboration (1); Others (4)	Residents (3); Nurses (3); Physicians (1); Students (5); Doctors (1); Surgeons (1); Anaesthesiologists (1)	Mixed-method (5); Quantitative (3); Qualitative (2); n/a (1)	Observational (5); Outcomes (2); n/a (5)	Unit (2); Department (4); Institution (3)	Second Life, Linden Research Inc. (1); iStan, Medical Education Technologies (1); ISEE Simulator, E-Semble (1); PediatricSim, Saint Louis University (1); First2Act, Monash University (1); SIMAN, Rockwell Software (1); ProModel, ProModel (1); AnyLogic, AnyLogic (1); Others (1);
Misc.	[182,183,184,185,186,187,188,189,190,191,192,193,194]	Communication (11); Teamwork (5); Leadership (3); Decision-making (3); Coordination/collaboration (1)	Residents (2); Nurses (4); Physicians (3); Students (3); Doctors (2); Midwives (1); Paramedics (1); Others (1)	Mixed-method (6); Quantitative (4); Qualitative (2); n/a (1)	Observational (8); Outcomes (2); n/a (4)	Unit (4); Department (6); Institution (3)	METI, Medical Education Technologies (1); ECS, Medical Education Technologies (1); Swiss medium fidelity simulator, Laerdal (1); iStan, Medical Education Technologies (1); PROMT flex model, Limbs and Things (1); ETS, Centre for Teaching and Research in Disaster Medicine and Traumatology (1); MACSIM, MACSIM (1);
Not specified	[24,195,196,197,198,199,200,201,202,203,204,205,206,207,208,209,210,211,212,213,214,215,216,217,218,219,220,221,222,223,224,225,226,227,228,229,230,231,232,233,234]	Communication (31); Teamwork (18); Leadership (12); Decision-making (12); Coordination/collaboration (2); Others (3)	Residents (4); Nurses (14); Physicians (8); Students (5); Doctors (10); Midwives (2); Therapists (1); Paramedics (7); Assistants (3); Surgeons (4); Anaesthesiologists (1); Technicians (1); Midwives (2); Others (9)	Mixed-method (13); Quantitative (14); Qualitative (12); n/a (4)	Observational (17); Outcomes (8); n/a (18)	Unit (19); Department (6); Institution (10)	EART simulation, Boston Medical Center (1)
Methodological contribution	[13,14,235,236,237,238,239,240,241,242,243,244,245,246,247,248,249,250,251,252,253,254,255,256,257,258,259,260,261,262,263,264,265,266,267,268,269,270,271,272,273,274,275,276,277,278,279,280,281,282]						
Literature review	[283,284,285,286,287,288,289,290,291,292,293,294,295,296,297,298,299,300,301,302,303,304,305,306,307,308,309,310,311,312,313,314,315]						

### 3.2. Communication 

Communication plays an important role in developing therapeutic relationships between patients. The ability to build trust in the process of achieving common goals has become a highly sought-after non-technical skill in emergency medicine. It is often directly linked to the proficiency of both verbal and non-verbal responses. This management of challenging interactions between individuals is essential nowadays in the catchment area of emergency departments and medical workplaces. Joined by approximately 600 students at a U.S. dental school, McKenzie et al. studied the social determinants of health through simulation exercises on communication [45]. Results confirmed clinical performance improvement thanks to communication skills training with standardised patients and mannequins. Ayub et al. investigated the experience of prehospital providers in simulation settings [64]. The authors concluded that communication could overcome the environment- and manpower-related barriers, which have emerged as major themes in the delivery of patient- and family-centred care. Communication skill performance can be used to assess the educational effectiveness of medical simulators. To enhance acute care nurses’ ability to identify and treat patient deterioration in a timely manner, Ozekcin et al. applied a non-technical skill framework in simulation exercises with debriefings [125]. Participants’ responses showed a broadened knowledge base and decreased time span to critical actions once they adopted the situation, background, assessment, and recommendation strategies to recognise the instability of patients.

### 3.3. Teamwork

Teamwork skills in medical simulations are pursued in many innovative programmes to close performance gaps that impair practitioners’ capacity. For teamwork training efforts to be successful, staff members must take part in the disclosure of urgent medical events and put forth a free flow of ideas across the emergency department. Teamwork could be exercised in simulation with overall improvements in team involvement, confidence, and objective understanding of the Emergency Airway Response Team, as suggested by Tsai et al.’s simulation project [210]. Knowledge assessment protocols might account for one’s capacity to work in teams. Semler et al. analysed the effects of teamwork training with high-fidelity simulations, expert demonstrations of principles, and traditional didactics [73]. Expert-dependent, time-intensive simulation interventions offered positive effects on teamwork skills. Although individual skills improved as a result of simulations, according to Nelson et al. [217], the establishment of a positive atmosphere was not observed for the surgical wards. In addition to Nelson’s suggestions for simulation-based training to raise awareness of safety issues, efficient teamwork was found to lower levels of patient harm in treatment [46]. Medical simulations combined with a structured teamwork curriculum could apply crew resource management principles to exemplify logistical considerations for acute stroke care.

### 3.4. Leadership 

Leadership skills are increasingly recognised as strong determinants of peer relationships, workplace performance, and patient outcomes in emergency medicine. To promote a working environment steered by commitment, responsibility, and social order, leadership brings together personnel across disciplines to explore dynamic processes. In a simulation-based, randomised study, Weller et al. investigated information-sharing channels in an anaesthetic emergency [128]. The researchers discovered a link between information-probe sharing and callouts. A proactive team leader may expedite the development of a shared mental model among team members. Team debriefing immediately after resuscitation in simulation-based sessions contributed to declaiming leadership on-scene with the ambulance crew, as suggested by Clarke et al.’s study [213]. It was not just fulfilling a dedicated role for someone to stay with certain tasks; the resuscitation team leader rotated through the scenarios in Ten Eyck et al.’s study [12], suggesting that simulation training was critical to further developing strong leadership in comparison to the group discussion format. Leadership can also be exercised in the interpretation of training outcomes from crew resource management courses—Parsons et al. incorporated medical simulations into a residency curriculum endorsed by SimMan 3G [60], showing evidence of effective simulation in the clear instruction of non-technical skills.

### 3.5. Decision-Making

Following the literature study, decision-making skills in emergency medicine provide practitioners with the capacity to react quickly and swiftly. The decision-making process in medical simulations emerges as a powerful problem-solving mechanism. In Morreel et al.’s study, the authors proposed a guideline for practitioners to determine patients’ triage urgency level in tandem with their present medical condition [183]. The simulation study confirmed the steps required for handling a telephone triage supported by the correct protocol, urgency category, and resource management. In addition, results showed acceptable performance of the decision support system. In Gerard et al.’s serious game study, participants acted as healthcare providers, assessing and treating a wide range of acutely ill patients [39]. Game scores can serve as valid indicators for assessing decision-making skills. In a randomised crossover study, Krage et al. identified declining non-technical performance once external stressors distracted individuals from routine tasks [120]. Their simulation study analysed the effects of stressful conditions on decision-making performance and technical skills training outcomes after a cardiopulmonary resuscitation scenario. Nicksa et al.’s interprofessional simulation simultaneously developed technical and non-technical skills when dealing with high-risk situations [74]. The recreated scenarios were realistic and beneficial to the individual proficiency of surgical residents before handling such circumstances in reality. To summarise what has been stated so far, self-correction, monitoring, adherence to guidelines, and interactions between team members are critical to the decision-making process of high-stakes emergency scenarios in medical simulations.

### 3.6. Coordination and Collaboration

The interest in coordination and coordination corresponds to a greater awareness of patients under the system. This is based on a thorough understanding of cross-departmental and cross-institutional delivery systems. When handling tasks for patients, such non-technical skills are warranted to offer appropriate recommendations. Abu-Sultaneh et al. used in-person simulations to activate collaboration within the regional hospital system [104]. The overall adherence to the critical action checklist increased to over 70% thanks to the quality management partnership. In a high-fidelity simulation study, Schumutz et al. explored the coordination of algorithm-driven and knowledge-driven implementations of emergency tasks [108]. It should be noted that coordination affected performance. Luctkar-Flude et al. evaluated the communication and interprofessional coordination skills of nursing and medical students after going through an asthma exacerbation simulation [83]. The design of medical simulation studies to practise interprofessional coordination skills was consistent with earlier research evaluating comfort levels and confidence with procedures. 

### 3.7. Others

Situational awareness, resource management, resource utilization, prioritisation, and time management were recognised as the less frequently trained non-technical skills in the medical simulation of emergency medicine. Here, situational awareness refers to the application of the personnel’s sensory systems to approach the working environment, bearing in mind anticipated threats and prospective consequences. Resource management addresses all logistical aspects of patient hand-off and transport. In light of this, resource utilisation emphasises the ability to reserve capacity in the occurrence of unexpected events. Prioritisation accounts for patient inflow mapping in which the most critically ill patients are given priority. Time management gives staff members back control over patients since emergency medicine would require simultaneous and sequential interventions performed by different personnel.

### 3.8. Scholarly Pattern Analysis

**Figure 1:**
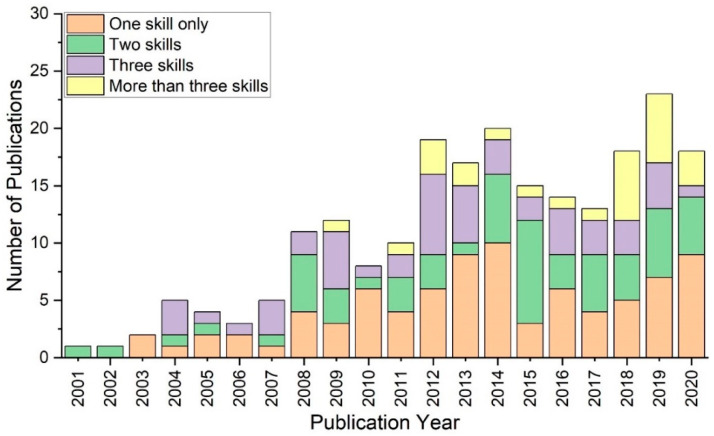
Number of non-technical skills in the literature between 2001 and 2020.

The literature study shows that approximately 50% of simulation studies conducted during the early years of publication featured only one type of skill. To date, this accounts for the greatest proportion across medical simulation categories. In contrast, only in 2008 and 2015 did the number of simulation studies involving two types of skills outnumber those of all other categories. As the number of simulation studies involving more than two types of skills has increased recently, this trend has been applied throughout the graph, as shown in Figure 1. After reaching 33% in 2018, the percentage of research requiring more than three types of skills decreased, with the lowest gap between the categories occurring in 2019. In 2020, more scholarly attention was devoted to simulation studies of only one type of skill and two types of skills.

**Figure 2:**
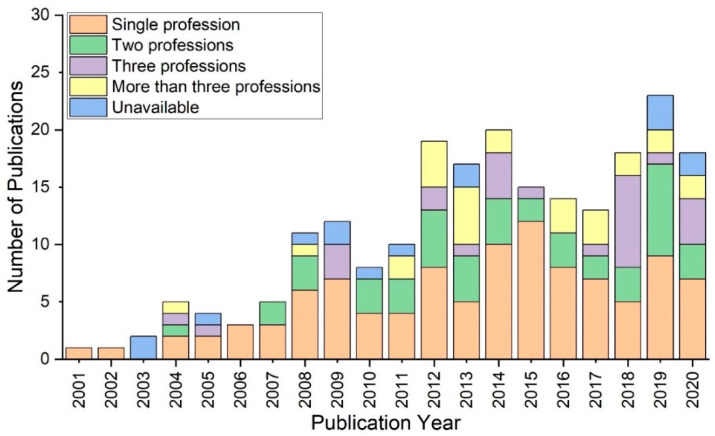
Number of studies involving professions between 2001 and 2020.

The bar chart indicates that simulation studies involving only one profession were distributed across the time period (Figure 2). While some researchers attested to the competency of two professions, others researched three or more. Overall, research involving only one profession had the largest number of publications, whereas there were few studies that did not explicitly state any profession. Those involving two professions and more than two professions had 59 and 76 publications, respectively. Notably, these categories have similar profiles. The frequency of publications involving only one profession grew substantially between 2001 and 2009. It fell somewhat between 2009 and 2010. Following that, the number of publications increased until it peaked at 12 in 2015. Its proportion, however, fell dramatically during the next five years.

**Figure 3:**
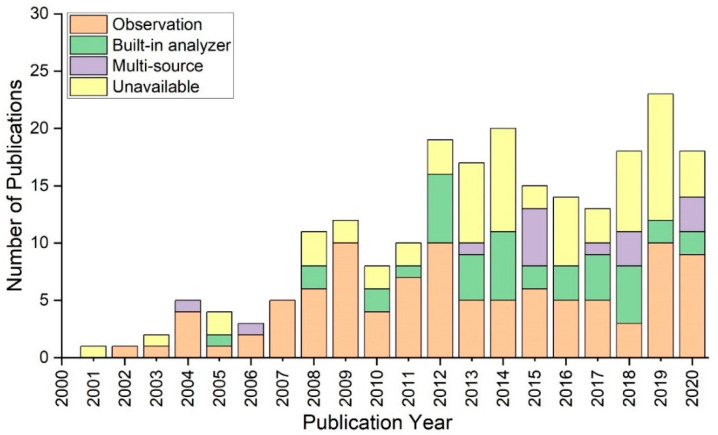
Number of studies interacting with behavioural data sources between 2001 and 2020.

Observations were the most common source of behavioural data during the time period, as shown in Figure 3. Between 2001 and 2010, the majority of research recognised video and verbal references among participants. In addition, built-in analysers have become more popular in screen-based and human–patient simulations. This was a promising source of behavioural data, whether quantitative or qualitative, as it provided a better understanding of the levels at which participants engaged the most. Simulation studies combining observational data and analysis procedures have contributed to a large proportion of publications between 2015 and 2020. However, in the last five years, studies that did not rely on any specific data sources have become more common. There is a need to take advantage of actionable intelligence in order to describe the psychological profiles of participants.

**Figure 4:**
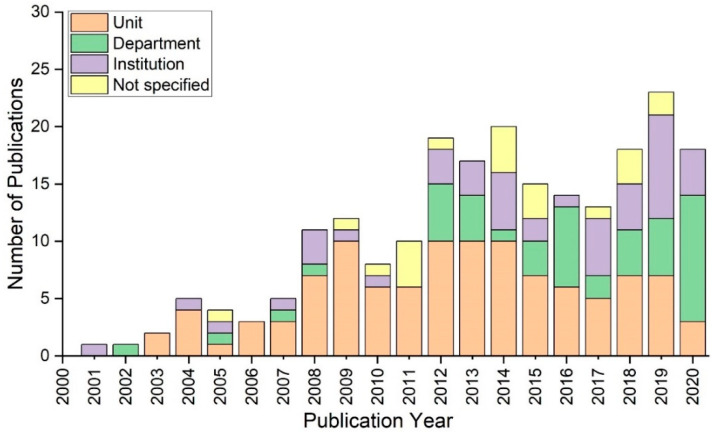
Number of studies carried out at the respective level of operational clinical environment between 2001 and 2020.

As seen in Figure 4, the literature study identifies the operational clinical environment at the unit, department, and institution levels. The majority of research in this sector has been unit-wide, carefully monitoring the health status of patients. The data reveals that 2009 was the year with the highest number of unit-wide simulation studies, followed by a gradual increase in the number of publications addressing non-technical skill issues at the institutional level. Prior to 2011, there was department-wide training, with a steady growth in publications from 2017 to 2020. It is important to note that a number of studies have not explicitly stated the level of the operational clinical environment. 

**Figure 5:**
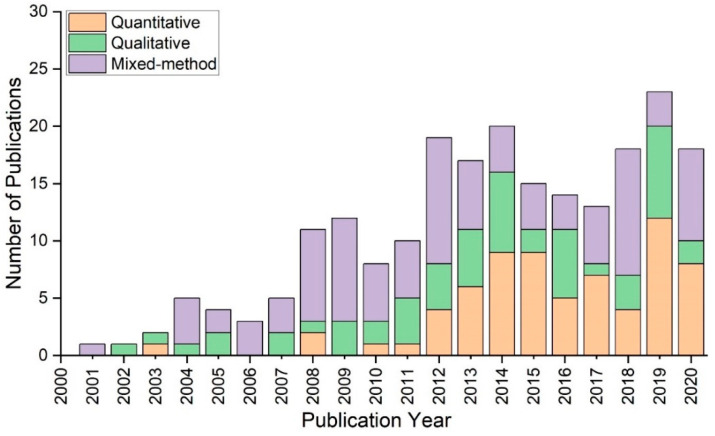
Number of studies employing quantitative, qualitative and mixed-method approaches between 2001 and 2020.

Figure 5 presents the number of papers employing quantitative, qualitative, or mixed-method approaches. More than half mixed-method studies were conducted before 2010. From 2011 through 2014, there was a small decline; however, in 2015, the number of articles using quantitative approaches grew to 9. After reaching its maximum, the number of publications declined to 8 by 2020. In 2007, only a couple of studies used qualitative methods. The number climbed to 7 in 2014 and then decreased dramatically in 2017. In the last three years, the number has varied from 8 in 2019 to 2 in 2020.

### 3.9. Bibliometric Analysis

**Figure 6:**
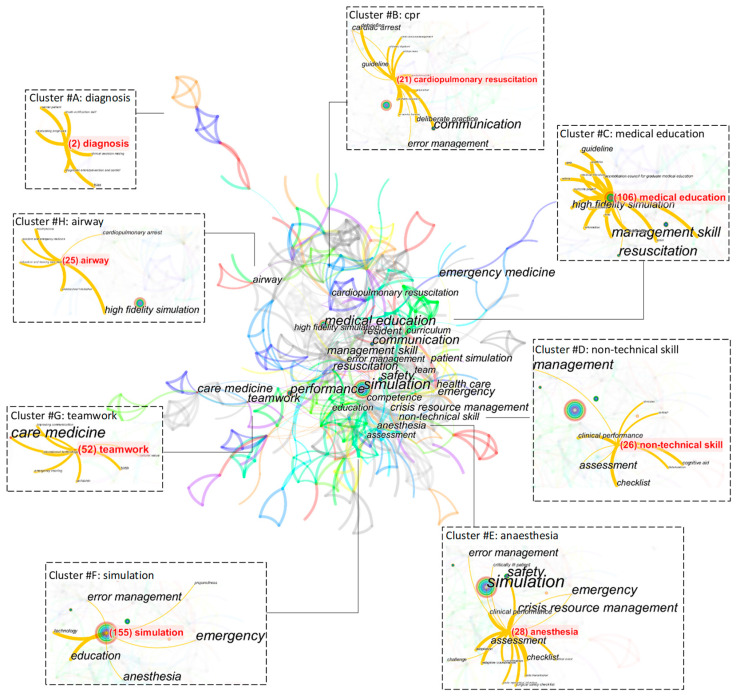
Cluster view of keyword concurrences between 2001 and 2020.

As shown in Figure 6, the citation networks of articles are formed by keywords such as simulation (155), medical education (106), teamwork (52), anaesthesia (28), non-technical skill (26), airway (25), cardiopulmonary resuscitation (21), and diagnosis (2). Remarkably, each network contains publications throughout the course of the time period. Simulation, the keyword from the core thematic trend, leads to technology, education, anaesthesia, error management, emergency, and preparedness—this is an example of how the central citation network has extended to spearheading research areas. Through research efforts on educational technology, communication, and cultural value, articles on teamwork have covered both the technological and social aspects. Up to this point, these networks have had a lower concentration. In contrast, medical education develops a more complex cluster despite being referenced less often. The second-largest cluster publishes articles in certain areas, such as high-fidelity simulation, management skills, guidelines, and resuscitation. This pattern also applies to other clusters with smaller numbers of publications. Anaesthesia is an important subcategory of study that led to the research fields of crisis resource management and safety. Similarly, non-technical skills, connected by clinical performance, assessment, and checklists, proceed to cognitive aid. Airways and diagnosis have created new research revenues in clusters away from their central counterparts, indicating a broader scale of medical simulations. 

**Figure 7:**
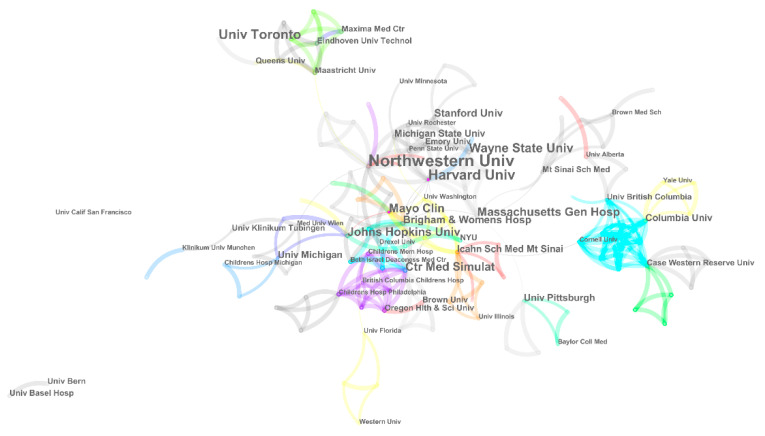
Cluster view of institutional collaborations between 2001 and 2020.

Figure 7 highlights institutional collaborations to illustrate the influence of academic centres searching for new areas. The visualisation consists of 58 nodes and 112 links during the time period. It is clear from the graph that Northeastern University and Harvard University are the most frequently collaborating partners in research activities. Particularly, several renowned institutions serving as guiding hands—the Medical Simulation Centre, Columbia University, and Case Western Reserve University—have contributed to high-level research collaborations as part of a thriving international community. However, growing networks joined by European affiliations have recently piqued research interests. To summarise what has been stated so far, remarkable collaboration networks are gathered through universities, hospitals, and labs in North America.

**Figure 8:**
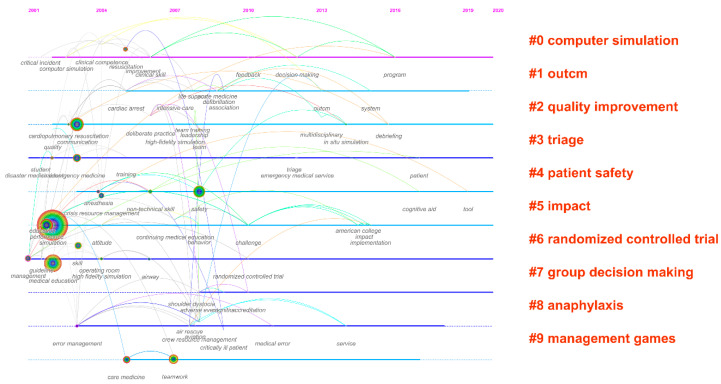
Timeline view of research thematic horizons and keyword changes between 2021 and 2020.

Figure 8 presents the timeline view of the CiteSpace map. The solid lines denote thematic horizons such as computer simulation, outcome, quality improvement, triage, patient safety, impact, randomised controlled trials, group decision-making, anaphylaxis, and management games, covering methodological and application-oriented perspectives. In addition, a group of phrases are identified as keywords to illustrate how they have changed over time. It is worthwhile to note that triage, impact, and randomised controlled trials exist throughout the time period. Prior to 2010, a preponderance of new ideas and approaches was created, as seen by the graph. The treatment of critically ill patients with anaphylaxis was inspired by the intersection of computer simulation and resuscitation. Simulation in the management game’s thematic horizon continues with medical care and teamwork. Last but not least, later in the timeline, there are keywords such as “cognitive aids,” “tools,” “patients,” and “programmes” on patient safety, triage, and computer simulation. 

### 3.10. Ring Model

**Figure 9:**
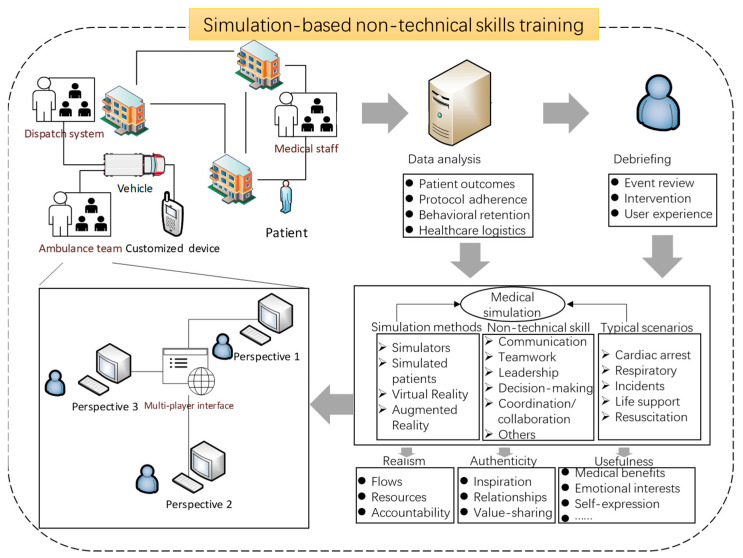
Ring model in the intersection between medical simulation, non-technical skills training and emergency medicine.

The literature study discovers no theoretical frameworks to synthesise previous research findings. To shed light on the linkages between medical simulations and experience design, which are promising to fertilise innovative ideas in both directions, this study presents a ring model as an integrated research framework (Figure 9). Amongst other things, debriefing is recognised as the main component to safeguard intended training goals, eliminate contextual limitations, and facilitate medical knowledge transfer to real working environments. It might be challenging to raise adjustments at each stage of the medical simulation study while also undergoing all stages of the ring model. However, these steps are necessary to concretise training outcomes in emergency medicine. In a nutshell, a medical simulation project is administered on the basis of all the design considerations for functionally driven and effectively organised non-technical skills training activities. 

The diagram shows the five stages of instruction that are given to the participants. According to prior studies, realism in the initial stage directly links to the description of patient flows, resources, and accountability frameworks. Participants engage in medical simulations only if they receive a sense of authenticity from the constructs, value sharing, and inspirations between team members. Simulation methods include simulators, simulated patients, Virtual Reality, and Augmented Reality, depending on the learning outcomes and objectives. Amongst other things, simulated patients are ideal for teaching communication, conflict management, compassion, and ethical dilemmas, whereas human simulators may be used to build therapeutic resilience. At this point, it is worthwhile to justify the application of simulation in terms of medical benefits, emotional interests, and self-expression. In light of this, data collection refers to gathering patient flow data, in accordance with the ethical agreement, to populate the simulation model. This is necessary to represent complicated and difficult cases. In the following stages, interprofessional teams are prepared for emergencies in a simulated setting. This is accomplished through mastering non-technical skills. Next, a data analysis is conducted using predetermined measures for patient outcomes, protocol adherence, behavioural retention, and healthcare logistics. The debriefing stage involves thought-provoking discussions in an iterative and retrospective process where team members learned from each other and from themselves.

If manipulators are employed, computer-assisted instruction has to be taken into account. The presence of engineers is also required. It is necessary to promptly and sensibly alter the physiological value in the teaching plan in response to the participants’ performance as per the real circumstances in the classroom. The instructor may utilise role-playing approaches to teach empathy or ethical issues. By role-playing, after-views and debriefing, this ring model could accommodate the learning process.

The debriefing stage involves thought-provoking discussions in an iterative and retrospective process where team members learned from each other and from themselves. Various debriefing provisions might be subject to concerns that interruptions disturb fidelity and adversely affect learning. The author suggests a rigorous comparison of debriefing strategies with medical simulations to explore the effects on non-technical skills acquisition and learning experience.

## 4. Discussion

This section describes the high-yield topics for future research. The answers to these research questions will inform medical simulation instructors on how to appropriately organize training content and handle design issues.

### 4.1. Practice-Based Training and Improvement

The context brought to interprofessional situations can influence the degree of contact and the pace of training. It is vital to have an overview of each team member’s skills and interests in order to respect altruism, culture, age, and gender. When aligning intended training goals, the first aspect that can be noted by instructors is their impact on the personnel undertaking interprofessional responsibility in medical simulations. A difference was made between the distribution of responsibilities among team members and the knowledge exchanges, according to MacNaughtonet et al.’s study [316]. In addition to the fundamentals of the medical system, participants need a comprehensive understanding of the activities and necessary skills to deliver excellent patient outcomes as the situation deteriorates. Even though patient scenarios could be supplied with task trainers [317], increasing the use of simulation in emergency medicine should allow the integration of skills that not only strives for professionalism but also clarify accountability frameworks in a dynamic process. 

Although delivering quality and safe care within the purview of a single institution is of primary concern, it is also encouraged for networked medical institutions to approach more complex issues with interdependent threads. The majority of research has focused on non-technical skills training in a clinical environment with limited operational capacity. Nevertheless, multi-centred simulation initiatives have been conducted in an effort to establish stronger cross-institutional partnerships [46,103]. The delivery of training experiences is on the rise to promote the innovative use of information technology with a focus on system solutions. Instructors may now elicit hitherto splendid and overlapping processes into complete medical procedures and patient management plans. In addition, cross-institutional comparisons of training results may be undertaken to analyse the areas that need to be improved in individual practice projects. This study posits the following question to be answered in future simulation projects:

- ‘How can various agencies be brought together via simulation-based non-technical skills training?’
- ‘How can medical simulations foster innovative strategies for addressing challenges that cannot be resolved independently?’

### 4.2. Teaching Theories and Educational Planning Models

Seldom did authors address teaching theories and educational planning models. However, it is becoming more essential to figure out how individuals receive, process, and retain information throughout learning. Kolb’s Learning Cycle [318], also known as the Experiential Learning Cycle, could serve as the cornerstone for a learning-by-doing paradigm. According to the framework that is primarily used in nursing education [319], concrete experience, reflective observation, abstract conceptualization, and active experimentation are the four stages that must be completed for successful learning. Also, further study may broaden educational planning models for the development of practical courses. One such template could be Kern’s six-step approach to clarify the intended learning outcomes of the overall programme and of individual sessions [320,321]. In such cases, the effective blending of the experience of learners together and instructional design is a keyword. The following research question should help researchers in the pursuit of a leaner-centred style:

- ‘How do we apply teaching theories and major approaches to educational planning with medical simulations for non-technical skills training in emergency medicine?’

### 4.3. Medical Simulation Verification and Validation

Although certification in medical simulations is becoming more of a priority to identify best practices for a wide range of providers and applications [322], the credibility of medical simulation models remains largely underexplored. Barnes and Konia confirmed many different interpretations and accomplishments in healthcare simulation studies [323]. For example, Abir et al. developed a discrete-event simulation model to predict capacity bottlenecks at a medical centre [176], allowing for improved decision-making with changes in the working environment. However, rarely have such reviews been conducted in medical simulations—the majority of articles recreated tasks, cases, and scenarios rather than closely examining logistical aspects of emergency medicine. Most medical simulation studies currently lack an iterative model-building procedure. This warrants validation and verification throughout the operational requirements. The following research questions are outlined in order to ensure an accurate description of the medical system:

- ‘What paradigms should be developed for verifying and validating medical simulations in emergency medicine?’
- ‘How can instructors describe the logistical aspects of emergency medicine using real-time clinical data and patient inflow patterns?’

### 4.4. Applied Ergonomics

Medical equipment and apparatus used with simulators should take ergonomics and human factor engineering into account. However, the literature study reveals inadequate attempts to reduce fatigue, discomfort, and injury. Such aspects of applied ergonomics may be enhanced to reduce participant risk and better-fit training processes. This is a promising research domain since medical equipment must frequently be manoeuvred quickly and safely in different simulated scenarios. 

Repeated exposure to high-fidelity simulations has turned out to be an effective teaching strategy [324]. It was shown that early exposure to a complex clinical environment was helpful for transitioning knowledge into practice [325]. Nevertheless, prior research has not demonstrated a correlation between repeated exposures and an increase in participant stress levels [326]. When the participant takes a considerable amount of experience in simulations, the relationship between the quantity of practice, the acquisition of non-technical skills, and the impact on the sense of well-being should be elucidated from an ergonomic perspective.

Aside from quantitative research analysing the distributions of cognitive stress, it is promising for qualitative studies of focus groups to look for patterns or themes across entire datasets. Furthermore, mixed-method studies may help with data interpretation and broaden the generalizability. Therefore, it is worthwhile to answer the following question:

- ‘How could technology-driven tools as user-centric design approaches overcome ergonomic issues with medical simulations?’

### 4.5. User Interface Design

In contrast to interactions with a conventional graphical user interface, which mostly involve using a mouse and keyboard, the next step in the evolution of user interfaces will make user interaction appear enjoyable, motivating, and intuitive. Researchers attempted scoring systems to measure multitasking skills in an emergency department [327] and address logistical bottlenecks in paediatric emergency medicine [328]. However, the literature study identifies only a few simulation projects that touched upon design issues. Due to a dearth of in-depth studies on user engagement and adoption, there is a lack of understanding of the expected actions from digital features. Designers should embrace such elements to improve the timeliness of task completion, reduce omissions, and pinpoint aspects of the user experience. 

The user interface design will serve as a key component of next-generation simulators. This tendency requires the optimum usability, performance, and manoeuvrability of the digital media objectives. To date, the design patterns used in medical simulations have not been elucidated. Not only can user interface elements behave in specific ways, but there are also visual aesthetics, inclusion, and the adoption of habit-forming technologies, all of which contribute to a higher level of authenticity. Despite these promises, the interface needs to be suitable for medical system integration [329]. Given the lack of a detailed examination of user interface design, the following research question needs to be investigated:

- ‘What is the cutting-edge innovation in interface design for the integration of function and usability?’

## 5. Conclusions

This literature study presents a synthesis of previous simulation projects that explicitly trained non-technical skills in emergency medicine. Over the decades, advances in a wide range of medical simulation categories have been made using scientific research evidence and statistical methods. These contributions addressed the intersections between medical simulations, non-technical skills training, and emergency medicine. In addition, the literature study reveals a substantial body of evidence-based practice that improves the design of training experiences for communication, teamwork, leadership, coordination, and decision-making. The presence of patient cases and the involvement of professionals showcase how the prior research design could be extended to more detailed patient scenarios. More importantly, simulation-based education should be considered as a teaching approach to substitute high-risk, rare, and complex situations in technical or situational simulations. In light of this, the ring model as an integrated framework is proposed for conceptualising the innovative design method—it aims for the transfer of simulation-based training outcomes to a real working environment.

It has been over two decades since the publication of the landmark report ‘To Err Is Human: Building a Safer Health System’ [330]. The growing awareness of medical mistakes is steering interest in teaching not just technical but also non-technical skills, an area of study that has received relatively little attention in general and in emergency medicine in particular. The medical system should be rethought as a social-technical system with processes and interactions across the medical pathways.

The literature study is subject to limitations. First of all, to avoid overlooking significant practice-based evidence, the search terms should combine keywords pertaining to the less often trained non-technical skills and more specifically employed research methods, respectively. In addition, despite using entries from the Web of Science Core Collection editions, the electronic data search may not be exhaustive. The omission of abstracts, conference proceedings, posters, and book reviews might lead to a selection bias in publications. Furthermore, the research only traced papers published after 2000. Attention to earlier articles may aid researchers in explaining the current status of the topic and pointing out a number of changes in the way the field is pioneered. In order to dive into the specifics, a previously proven methodology for profiling key variables in healthcare simulations [331], explored by earlier researchers, would benefit from visualising the design of queries, the use of search tools, and the evaluation of each article. Further literature studies shall reflect on lessons learned from the gradual shift towards competency-based medical education over the past decades, the meaningful presence of milestones and entrustable professional activities in medical behaviour [332], and how simulations in general have built surrounding heuristics into medical education. 

Since the literature study is pioneering in this area, a synthesis of the outcomes over the past two decades outlines a broad array of research questions on the implementation of medical simulations in general and non-technical skills training in emergency medicine in particular. The need to capitalise on the characteristics of medical simulations warrants further investigations on practical training, validation and verification of medical simulations, user interface design, and applied ergonomics. These reflections on emerging research avenues should contribute to the body of knowledge on these underexplored areas. It is anticipated that future research will continue to deliver user-centric training experiences but will undertake an innovative design approach dedicated to the pursuit of performance excellence in simulated settings and longer retention of skills after completing the assignment. Last but not least, from a methodological standpoint, more qualitative studies should be carried out to streamline discourses from verbal, written, or language-use materials. Particularly, the application of thematic and content analysis, which has been largely unexplored in prior research, will help close the knowledge gap in the achievement of training goals and the provision of training experiences.

## Data Availability

Data used to support the findings of this study are available from the corresponding author upon request.
